# A New Approach in Prebiotic Chemistry Studies: Proline Sorption Triggered by Mineral Surfaces Analysed Using XPS

**DOI:** 10.3390/life13040908

**Published:** 2023-03-30

**Authors:** Eduardo J. Cueto-Díaz, Santos Gálvez-Martínez, María Colin-García, Eva Mateo-Martí

**Affiliations:** 1 Centro de Astrobiología (CSIC-INTA), Ctra. Ajalvir, km. 4, Torrejón de Ardoz, 28850 Madrid, Spain; 2 Instituto de Geología, Universidad Nacional Autónoma de México, Ciudad de Mexico 04510, Mexico

**Keywords:** proline, XPS, infrared, prebiotic chemistry, mineral surfaces, montmorillonite, olivine, haematite, iron disulphides, spectroscopies

## Abstract

The role of minerals in the origin of life and prebiotic evolution remains unknown and controversial. Mineral surfaces have the potential to facilitate prebiotic polymerization due to their ability to adsorb and concentrate biomolecules that subsequently can catalyse reactions; however, the precise nature of the interaction between the mineral host and the guest biomolecule still needs to be understood. In this context, we spectroscopically characterized, using infrared, X-ray photoemission spectroscopy (XPS) and X-ray diffraction (XRD) techniques, the interaction between L-proline and montmorillonite, olivine, iron disulphide, and haematite (minerals of prebiotic interest), by evaluating their interaction from a liquid medium. This work provides insight into the chemical processes occurring between proline, the only cyclic amino acid, and this selection of minerals, each of them bearing a particular chemical and crystal structures. Proline was successfully adsorbed on montmorillonite, haematite, olivine, and iron disulphide in anionic and zwitterionic chemical forms, being the predominant form directly related to the mineral structure and composition. Silicates (montmorillonite) dominate adsorption, whereas iron oxides (haematite) show the lowest molecular affinity. This approach will help to understand structure-affinity relationship between the mineral surfaces and proline, one of the nine amino acids generated in the Miller-Urey experiment.

## 1. Introduction

Minerals have been considered the key player for prebiotic synthesis [1]. Indeed, nowadays, researchers have confirmed the catalytic properties of many prebiotic mineral catalysts towards the nitrogen and carbon fixation reactions, and based on these features, it is evident that minerals have actively participated in the availability of some key elements on modern and primigenial Earth.

Minerals are conspicuous components of environments where chemical evolution could have occurred, for example, meteorites, comets, the surface of early Earth and other planets. Minerals have been proposed as key elements in prebiotic chemistry studies and could have behaved as catalysts to favour prebiotic chemical reactions [2].

The catalytic abilities of metals have been thoroughly recognized [3]. Biochemistry is extremely linked to metals, metabolism is driven by metal gradients, and they are part of the active sites of enzymes [4]. In fact, one important fraction of known proteins (almost one-third) contains metal cofactors [5]. This link is probably rooted to the environment where life originated [4], and it has been proposed that metal ions could be the first catalyst during the first stages of the Earth [6]. Keeping this in mind, there are studies interested in elucidating the role of metals in prebiotic environments. For example, Pinter et al. (2021) [7] demonstrated that transition metals (Zn^2+^, Cu^2+^y Co^2+^) increase the yield of depsipeptides, which contain histidine. In another study, Muchowska et al. (2017) [8] reported that some non-enzymatic reactions of the rTCA (reverse Krebs cycle) were promoted by metals (Zn^2+^, Cr^3+^ and Fe^0^). Bray et al. (2018) [9] studied the structure, function, and cation content of ribosomes under conditions of the early Earth; they demonstrated that iron and manganese (Fe^2+^ and Mn^2+^) are essential cofactors for the structure and function of ribosomes. Minerals could have also behaved as catalysts to expedite the release of chemical energy in water–rock–organic systems, enabling prebiotic chemical reactions [10]. It has been proposed that metal-containing minerals could be the predecessors of enzymes. There are resemblances in both the structure and reactivity of metallic minerals and the active sites of some enzymes [11]. In fact, most reactions involved in the bioenergetics of living beings require the participation of metal clusters in metalloenzymes [12].

Mineral surfaces have the potential to facilitate prebiotic polymerization by adsorbing and concentrating precursors or organic molecules on their surface (for example, Zaia et al., 2012 [13]; Rimola et al., 2019 [14]), which may result in catalytic reactions; however, the precise nature of the interaction between the mineral host and the biomolecule guest must be deeply studied. Over 70 years ago, it was proposed by Bernal [15] that the polymerization of biomolecules actually involved reactions on mineral surfaces, a mechanism later dubbed “polymerization on the rocks” [16,17]. For a long time, few experimental investigations were undertaken to test this hypothesis, so the adsorption and reactivity of amino acids and “small peptides” and their subsequent reactivity on surfaces resembling minerals that were probably present on the early Earth, such as iron disulphides, clays as montmorillonite, silicates as olivine, and iron oxides as haematite, would be a relevant approach to this hypothesis.

**Figure 1:**
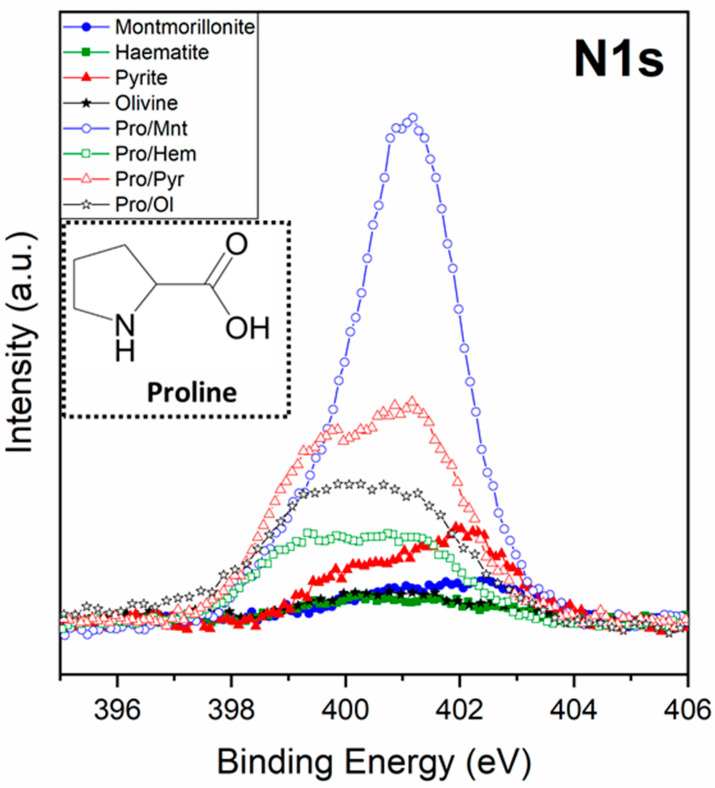
XPS nitrogen signal of the four minerals, before and after the adsorption of proline on montmorillonite, olivine, haematite, and iron disulphide; inset of proline molecular structure.

Amino acids are the building blocks of proteins, and they are essential units to build up more complex molecules from polymerization reactions [18,19]. Among all the amino acids, proline (C_5_H_9_NO_2_) is the only amino acid lacking primary amine groups; in fact, the amine group is located inside the heterocyclic-pyrrolidine group (see Figure 1). In proline, the carbonyl group is more electron-rich than those of other natural amino acids. This fact promotes the formation of hydrogen bridges, which are involved in macromolecular self-organization and currently play an important role in protein structure and function.

Proline is the only cyclic amino acid, and this molecule is of particular interest because it contains a secondary amine (NH) group, fixed rigidly in the pyrrolidine ring, limiting the conformational mobility of the N–H bond with respect to the carboxyl group (COOH), which is itself restricted in mobility by the pyrrolidine cycle [20,21]. This rigidity is one of the factors that determines the important role played by proline in the folding of proteins and peptides, where steric effects arising from the proline side chains help to determine the stabilities and positions of the protein folds [22]. In modern living beings, proline-rich regions (PRRs) are common in both eukaryotic and prokaryotic proteins [23], and these PRRs are involved in protein–protein recognition processes [24]. Furthermore, proline has been synthesized in prebiotic experiments under different conditions [25,26,27,28,29,30]; it has also been detected on meteorites [31,32].

Therefore, in this complex context, these experiments are focused on understanding how an amino acid, proline, interacts with different mineral surfaces (relevant in prebiotic chemistry) due to their different functionalities or capacities. Surface science tools such as XPS have been used to study pyrite surface-driven molecular chemistry [33,34], and understanding the reactivity of molecule-pyrite surface systems has been improved by using a wide range of studies characterized by powerful and innovative spectroscopy techniques. These techniques have perhaps been the most useful in establishing the structure of the pristine pyrite surface and molecule/pyrite interaction. However, despite their importance, few spectroscopic studies have been performed on molecular interactions on other mineral surfaces. It is well known that the adsorption of organic molecules or biopolymers on mineral surfaces may substantially modify mineral morphology and surface composition and reactivity, thereby tailoring their properties [35]. Therefore, surface science techniques are very promising tools to study these molecular processes on catalytic mineral surfaces to obtain molecular information.

On the early Earth, ultramafic rocks were very abundant. On these rocks, olivine and pyroxene were the most common minerals [36], and this lithology probably hosted hydrothermal systems [37]. Olivine is not a mineral species but a group of them, and the olivine group corresponds to a solid solution with a variable chemical composition (Fe_1−x_Mg_x_SiO_4_) [38], in which the Fe end member is called fayalite and the Mg end member is forsterite. The Earth crust is composed of more than 60% olivine, and fayalite is the most abundant mineral in the mantle [39].

Clays were the first minerals employed in prebiotic experiments since the proposal of Bernal [15]. Clay minerals are complex layered aluminium hydrous silicates. One sheet is formed by tetrahedral silicates, and the other by octahedral hydroxide [40]. The sheets are linked together by hydrogen bonds, and their assembly determines the type of clay, for example, 2:1 clays (two tetrahedral and one octahedral) or 1:1 clays (one tetrahedral and one octahedral) [40]. Montmorillonite is a 2:1 clay and is the most commonly used clay in prebiotic experiments; between the 2:1 blocks, there is a channel where cations can be stored and released.

Pyrite (FeS_2_) is a very common mineral on Earth [41] because it is formed by different geological processes; it can be found in sediments [42] or as a result of hydrothermal activity [43]. On our planet, pyrite allows many geochemical and biogeochemical processes [44], and it has been proposed as crucial in the iron-sulphur world hypothesis [45]. Due to its properties, it has also been used in many prebiotic experiments [33,35,46,47,48]. It has been demonstrated that pyrite can act as a catalyst for nitrogen fixation [49]. Pyrite and haematite are common minerals in hydrothermal environments [50].

The potential catalytic activity of minerals in the context of prebiotic chemistry remains for the most part unexplored. In this scenario, we have studied the interaction of proline with montmorillonite (Mnt), olivine (Ol), iron disulphide, and haematite (Hem) by evaluating the interaction of the amino acid in a liquid alkaline environment. This strategy was designed to evaluate how mineral compositions modify the molecular interaction between the amino acid and the iron, silica, and sulphur surficial groups of the minerals. The interaction of some functional groups in organics may be governed by electrostatic forces (physisorption), while the interaction of others may be dictated by chemical bonding (chemisorption) to a specific group on the surface. In the present work, we will try to respond to these uncertainties by applying spectroscopic techniques.

In our study, we apply X-ray photoelectron spectroscopy (XPS), which is a surface-sensitive spectroscopic technique that can identify the elements that exist within a material (elemental composition) or on its surface, as well as their chemical state (e.g., oxidation state or chemical group which belong to). XPS is based on the photoelectric effect, in which a free electron is ejected from an atom after it has absorbed the energy of a photon from a source (X-rays). The relationship between the kinetic energy of the free electron (Ek), the binding energy (Eb), the photon energy (hν), and the work function (Ǿ) can be expressed as:

Ek = hν − Eb − Ǿ(1)

Knowing the energy of the incident photon and measuring Ek, we can obtain Eb. The electron binding energy (Eb) is a parameter that identifies the electron specifically in terms of the element and atomic energy level from which it comes [51,52,53,54,55]. The energy emitted by the photoelectrons is analysed using a spectrometer, and the data are plotted on a graph of intensity versus binding energy. The number of detected electrons in each peak is directly related to the amount of element within the XPS sampling volume. XPS is a powerful measurement technique because it shows not only what elements are present but also what other elements they are bonded to.

The study of the physical properties of minerals is fundamental for understanding the chemical reactions driven by them. Furthermore, developing a picture (at the atomic level) of the molecular structure and reactivity of these surfaces is also critical to understanding the role of these surfaces in the formation of complex biomolecules in a broad environment.

## 2. Materials and Methods

### 2.1. Materials

Four different minerals were used for the experiments (see Table 1): Na-rich montmorillonite (SWy-3, County of Crook, WY, USA), chemical composition (%) SiO_2_: 62.9, Al_2_O_3_: 19.6, Fe_2_O_3_: 3.35, MgO: 3.05, CaO: 1.68; iron disulphide (>99.9%, Thermo Scientific^®,^ Waltham, USA); haematite (NCS DC^®^ 14033), chemical composition (%) SiO_2_: 9.82, Al_2_O_3_: 0.48, CaO: 0.11, TFe: 61.68, FeO: 1.43; and olivine–basalt RM No. 1044-94^®^. L-proline (≥99.9%) was purchased from Sigma–Aldrich^®^ (Missouri, USA).

### 2.2. Interaction Process/Adsorption Experiments

The adsorption experiments were conducted by using the same stock solution of L-proline (1 M; pH 10). None of the natural minerals or FeS_2_ were pre-treated or previously purified.

Ten millilitres of the solution of L-proline was mixed with 300 mg of Na-rich montmorillonite in a Teflon-capped reaction vial. Thereafter, the reaction was heated to 80 °C and stirred for 48 h. Then, the mixture was cooled down, and the collected suspension was centrifuged (13,000× *g* rpm, at RT). The supernatant was discarded, and the remaining solid was exhaustively washed with Milli-Q water (3 × 10 mL). The sample (Pro/Mnt) was dried under high vacuum, leading to 280 mg of a pale solid. In the case of the control sample, 10 mL of Milli-Q water was added to 300 mg of Na-rich montmorillonite and treated as previously described. Once the sample was dried under high vacuum, 282 mg of a pale solid was recovered.

The experimental protocol carried out for the Na-Montmorillonite (Mnt) sample case was repeated for the adsorption experiments in haematite (Hem), olivine (Ol), and pyrite (FeS_2_), recommended abbreviations for the names of clay minerals and associated phases [58]. The quantities of sorbate/mineral obtained after work-up were roughly similar, leading to approximately 280 mg in each case.

### 2.3. Characterization by XPS, IR, and XRD Analysis

The XPS spectra of the mineral samples were recorded before and after the interaction among the molecule, from the solution, and the mineral. The XPS analysis of the samples was carried out in an ultra-high vacuum chamber equipped with a hemispherical electron analyser and with the use of an Al Kα X-ray source (1486.6 eV) with an aperture of 7 mm × 20 mm. The base pressure in the chamber was 2 × 10^−9^ mbar, and the experiments were performed at room temperature. The peak decomposition in different components was shaped, after background subtraction, as a convolution of Lorenztian and Gaussian curves. Binding energies were calibrated against the binding energy of the carbon 285.0 eV samples.

IR analysis and Fourier transform infrared (FTIR) spectroscopy of the mineral and proline/mineral systems were performed on eight samples in a Thermo-Nicolet spectrometer. Spectra (2 cm^−1^ resolution and 128 scans) were collected in the mid-infrared region (400–4000 cm^−1^) using a DTGS-ATR detector and an XT-KBr beam splitter.

X-ray diffraction analysis and XRD characterization were carried out using a Bruker D8 Advance diffractometer with Cu Kα radiation (λ = 1.542 Å) and a diffractometer using Cu K1α radiation (λ = 1.54056 Å) operating at 45 kV and 40 mA. A Bragg–Brentano configuration geometry was used. The 2θ range was from 0 to 40 or 60° at 0.041 scanning steps.

## 3. Results and Discussion

In order to study the L-proline-mineral interaction, XPS analysis was performed. InXPS photons with sufficient energy are absorbed by a system, core electrons are ejected from the sample. A typical XPS spectrum is a plot of the number of electrons detected at a specific binding energy. Each element produces a set of characteristic XPS peaks. These peaks correspond to the electron configuration of the electrons within the atoms, e.g., core levels 1s, 2s, 2p, 3s, etc. Therefore, XPS allows confirmation through molecular fingerprints of sorption, and those traits would confirm the presence of proline on the mineral surfaces.

### 3.1. L-Proline Adsorption on Mineral Surfaces

#### 3.1.1. XPS Studies

First, it is relevant to search for the presence of nitrogen as a molecular fingerprint since it confirms the adsorption and, therefore, the presence of the amino acid on the mineral surfaces. Figure 1 shows the XPS spectra of the N1s core level of proline on all minerals (montmorillonite, iron disulphide, haematite, and olivine). In the four cases, a nitrogen signal was detected, which proves the adsorption of proline on all the minerals tested. Furthermore, the intensity of the signal is related to the amount sorbed, so we can conclude that proline has a higher affinity for montmorillonite, followed by iron disulphide (pyrite), whereas olivine and haematite show low molecular affinity. This behaviour would be in good agreement with the fact that exposure of clay minerals to alkaline fluids, as in our case, induces the dissolution of silica, which can also lead to an increase in the specific surface area [59], favouring an increase in molecular adsorption on the mineral. In addition, montmorillonite has an interlayer space that holds cations [60], which could behave as a specific cation binding site.

One of the main objectives of this research was to deeply understand the sorption process. This objective has to do, on the one hand, with the identification of the functional groups of proline that interact with each mineral and, on the other hand, with determining the state of the amino acid (for example, ion, zwitterion, neutral molecule) when adsorbed on each mineral. The proline molecule can exist in four possible chemical species, namely, neutral (molecular form), cationic (NH group is protonated to give NH_2_^+^), anionic (COOH group is deprotonated, leading to COO^−^), or zwitterionic (with both NH_2_^+^ and COO^−^ groups). The main factor that determines the chemical species (of an amino acid) present in a given system is the immediate geochemical environment. The zwitterion form dominates in the solid-state and in aqueous solution (near the isoelectric point, proline has a pKa_1_ = 1.99 (carboxyl), pKa_2_ = 10.96 (amino)). The anionic and cationic forms are pH-dependent and are abundant in alkaline and acidic media, respectively. The molecular form is present in the gas phase and can be isolated in a low-temperature argon matrix [61], while an anionic form, formed via the oxygen atoms of the carboxylate group and the nitrogen of the imine group, is predominant in the interaction on metallic surfaces such as copper [62].

**Figure 2:**
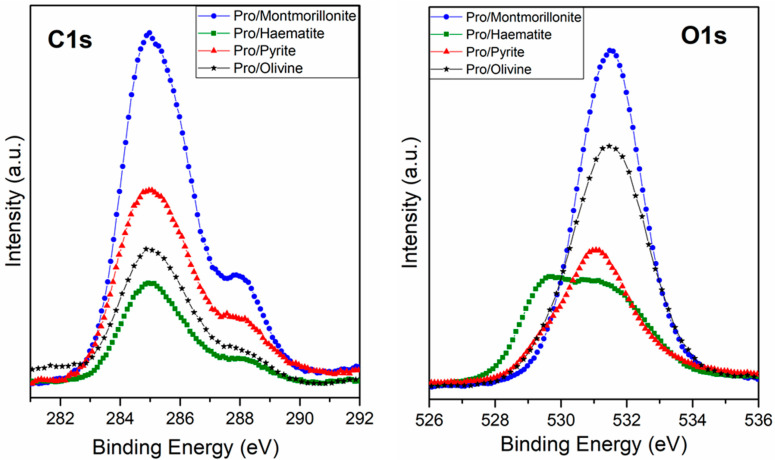
XPS spectra from the carbon (C1s) and oxygen (O1s) regions for the adsorption of proline on montmorillonite, olivine, haematite, and iron disulphide.

In our case, L-proline yields a clearly differentiated spectroscopic fingerprint on the core level spectra of N1s, C1s, and O1s, the latter of which are shown in Figure 2. The comparative study of the C1s core level spectra among different minerals confirms that L-proline has a high affinity for montmorillonite, whereas for iron disulphide, olivine, and haematite, it exhibits a lower molecular affinity. Moreover, although the intensity of the signal changes in each experiment, the profile of the spectra is similar for the four samples. In the case of the O1s spectra, the contribution of the mineral structure to this region of the spectrum needs to be considered; in particular, iron oxides, iron silicates, and iron sulphates (all oxygen-bearing minerals) confer relevant changes in the profile of the spectrum related to the chemical composition of each mineral under study.

A detailed analysis and deconvolution of the N1s, C1s, and O1s components confirm the molecular chemical form of L-proline adsorbed on each mineral surface.

The XPS deconvolution for the proline/montmorillonite system indicates that the best fit for the C1s peak presents three main contributions: the first, at 284.6 eV binding energy (B.E.), assigned to CH plus one adventitious component; the second (at 285.9 eV) related to the C-N groups; and the third one (288.1 eV) assigned to the carboxylate (COO^−^) or carboxylic (COOH) group. Regarding the O1s peak, the experimental data adjustment reveals the contribution of one component at 531.0 eV, which has been assigned to the two equivalent oxygen atoms belonging to the so-called resonant state of the deprotonated carboxylic group (carboxylate group), and the adventitious oxygen component due to the atmospheric air sample exposition before measuring XPS [63,64,65]. The N1s core level presents two components, the first one at 399.5 eV (19%) assigned to the NH species and the second centred at 401.2 eV (81%), which is assigned to the NH_2_^+^ species [66]. In summary, this spectroscopy analysis indicates that the molecule is adsorbed on the montmorillonite surface mainly in its zwitterionic (81%) and anionic forms (19%).

In the case of the Pro/Pyr system, the XPS analysis shows the same three components for the C1s peak at 284.2 eV, 285.9 eV, and 288.2 eV binding energies (B.E.), assigned to the CH plus adventitious carbon, to the C-N groups, and to the carboxylate (COO^−^) or carboxylic (COOH) group, respectively. In the O1s peak, two components were found, one each at 529.6 eV (assigned to the iron oxides) and at 531.0 eV, which has been assigned to the carboxylate group and an adventitious component, respectively [63,64,65]. The N1s core level also presents two components, at 399.5 eV (65%) assigned to NH species and the second at 401.1 eV (35%) assigned to the NH_2_^+^ species; accordingly, the proline molecule is present in different chemical forms, the anionic (65%) and the zwitterionic form (35%), and pyrite seems to enhance the anionic adsorption with respect to the zwitterionic form. Cystine also exhibits the same behaviour, and there is a preference of the anionic form to be sorbed on a pyrite surface, as described previously [46]. XPS analysis for the proline/olivine system shows the same three components for the C1s peak, which were observed at 284.5 eV, 285.9 eV, and 287.8 eV binding energy (B.E.) assigned to the CH and adventitious carbon, to the C-N groups, and to carboxylate (COO^−^) or carboxylic (COOH) groups, respectively. Regarding the O1s peak, two components were identified at 529.4 eV (assigned to iron oxides) and at 531.0 eV, which have been assigned to the deprotonated carboxylic group (carboxylate group) and adventitious oxygen. The N1s shows two components, at 399.5 eV (57%) assigned to NH species, and the second at 401.3 eV (43%), which is assigned to the NH_2_^+^ species; therefore, in this case, the molecule also presents two different chemical forms as well as in the case of pyrite. In both surfaces, the sorbed proline is predominantly in the anionic form over the zwitterionic form. However, in the case of iron disulphide, the percentage of anionic adsorption increases slightly with respect to the olivine surface.

XPS analysis for the proline/haematite system shows the same three components for the C1s peak at 284.7 eV, 286.0 eV, and 288.1 eV binding energies (B.E.). Those peaks can be assigned to the CH and adventitious carbon, to the C-N groups, and to carboxylate (COO^−^) or carboxylic (COOH) groups, respectively. The O1s deconvoluted spectra show three main components centred at 529.5 eV that can be assigned to iron oxides, at 531.2 eV assigned to the carboxylate group, and at 532.1 eV assigned to the hydroxide species. The N1s shows two components, at 399.3 eV (50%) assigned to NH species, and the second at 401.3 eV (50%), which is assigned to the NH_2_^+^ species[67]. In this case, L-proline is observed to be present in two different chemical forms, as in the previous cases (anionic and zwitterionic forms), but it is noteworthy that in the case of haematite, there are no preferential adsorption forms, meaning that both molecular forms are equally sorbed. Table 2 shows a summary of the proline species percentages adsorbed on each molecule/mineral studied system.

#### 3.1.2. FT-IR Studies

**Figure 3:**
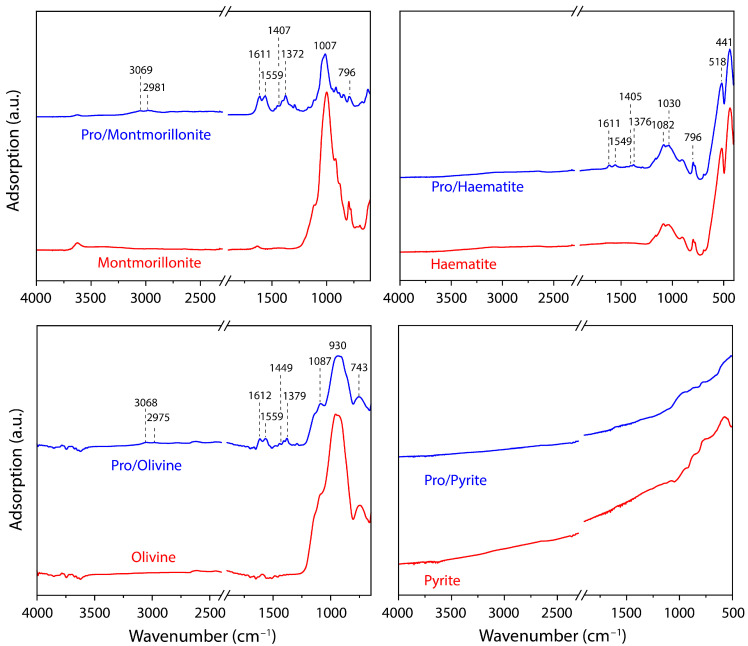
ATR-FTIR spectra for the minerals (red curves) and proline/mineral systems (blue spectra).

For a better identification of the vibrational groups and accurate confirmation of the molecular chemical forms adsorbed on the different minerals studied, the IR-ATR spectra of proline adsorption on the four minerals studied were recorded in Table 3, which shows the peak assignments. A careful analysis of the IR spectra was carried out in the 1200–1800 cm^−1^ region, where significant changes were observed between the mineral matrix and the mineral exposed to proline adsorption (Figure 3). Unfortunately, the dark surface of pyrite does not give a good reflection of the infrared beam, and therefore, we could not obtain a good signal in the spectrum.

The chemical forms of adsorbed L-proline may be distinguished by characteristic vibrational bands associated with the main functional groups present. The zwitterion form is identified on the four minerals studied by both the vibrations associated with the scissor deformation of the NH_2_^+^ group δ(NH_2_^+^), at approximately 1559 cm^−1^, and the asymmetric and symmetric carboxylate stretches, ν(COO^−^), at approximately 1611 cm^−1^ and 1402 cm^−1^, respectively. Due to the molecule/mineral interaction, the absence of the strong stretching vibration ν(C=O) at 1790–1760 cm^−1^, assigned to the COOH group, is noticeable, while the appearance of vibrations attributable to the COO^−^ functionality is evident [62].

For the proline anionic form, one can expect the presence of characteristic vibrations associated with COO^−^, as already described for the zwitterionic form. The NH-functionality vibrations (specific to the anionic form) are identified by the presence of the NH-bending vibration at 1377 cm^−1^, which is observed in the IR spectrum. Furthermore, vibrations assigned to the other molecular functionalities, such as ν(CH_2_)_sciss_ at 1452 cm^−1^ and ν(CH) bend at 1377 cm^−1^, were also identified. Additionally, the spectra show less intense bands in the 3100–2800 cm^−1^ region; nevertheless, it is possible to identify the presence of ν(NH) at 3060 cm^−1^, ν(CH_2_)_asym_ at 2981 cm^−1^, ν(CH_2_)_sym_ at 2947 cm^−1^, and ν(CH) at 2870 cm^−1^, which are also related to molecular vibrations from the proline molecule.

#### 3.1.3. XRD Studies

**Figure 4:**
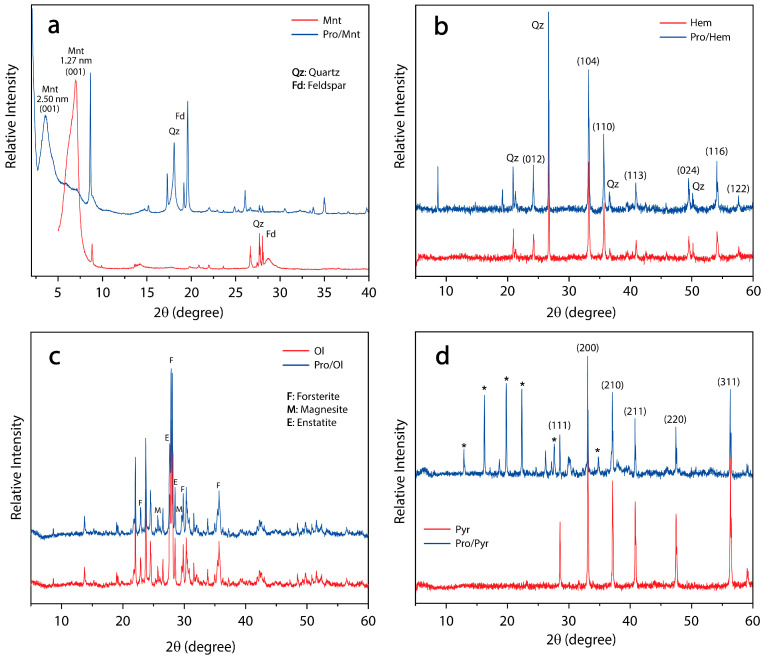
XRD diffractograms of the four cases of study: Pro/Mont (**a**), Pro/hem (**b**), Pro/Ol (**c**), and Pro/Pyr (**d**) in blue and their corresponding control samples in red. The measured diffraction peak positions and intensities are a fingerprint-like of a particular crystalline phase. Sharp and narrowed peaks at the XRD pattern indicate certain degree of crystallinity, whereas broad peaks denote amorphous materials, such as the (001) lattice plane in both montmorillonite cases.

The XRD patterns of montmorillonite, pro/montmorillonite, haematite, pro/haematite, pyrite, pro/pyrite, olivine, and pro/olivine powder are shown in Figure 4 (below). The diffractograms showed that montmorillonite (Figure 4a, in red) displays a typical diffraction peak at 2θ = 6.99°, corresponding to a basal interlayer distance of 1.27 nm according to Bragg’s law [74]. A second narrowed peak was observed at 2θ = 8.83°, which could be ascribed to the partial collapse of the mineral when exposed to pH = 10 (NaOH). The group of peaks observed between 2θ = 27–29° can be attributed to impurities of quartz and feldspar.

After the adsorption of L-proline (Figure 4a, blue), the interlayer distance (d_001_) increased to 2.50 nm. This result confirms that the organic molecule was profusely intercalated into the Mnt interlayer spaces. As the interlayer channel usually hosts cations, it can be suggested that the more likely proline species adsorbed here is the zwitterion form, while the sorption of the anionic form could occur on the surface of the clay.

On the other hand, both the haematite (Figure 4b) and olivine (Figure 4c) cases of study showed a similar pattern between the pristine (in red) and the mineral exposed to proline (blue). These results are in agreement with the small amount adsorbed on the surface recorded by XPS and FT-IR spectroscopies on the mineral surface.

Finally, the XRD diffractograms of the pyrite samples (Figure 4d) were recorded. As expected, the sample in the absence of proline displayed the typical pyrite pattern [75] showing six intensive peaks (111), (200), (210), (211), (220), and (311) (Figure 4d, red); however, due to oxidation, six new peaks (denoted with an asterisk) arise and are attributed to Rozenite (FeSO_4_ · 4H_2_O) formation. This is in agreement with the observed XPS spectra of pyrite (S2p). On the other hand, when pyrite is in the presence of proline, the oxidation of FeS_2_ is prevented. This is also confirmed using XPS spectroscopy show at the 3.2.2. Iron Disulphide section.

After IR and XPS analysis, we confirmed that the only two chemical species of proline adsorbed onto mineral surfaces are zwitterionic and anionic and that the percentages of each species are different depending on the nature of the mineral. Montmorillonite has a strong affinity for the zwitterionic form, whereas pyrite strongly interacts with the anionic form, whereas olivine and haematite barely discriminate each chemical species. A detailed mineral analysis of relevant elements such as iron and silicates would then be necessary.

### 3.2. The Role of the Mineral Surface in the Competitive Adsorption of Zwitterionic and Anionic Species

#### 3.2.1. Montmorillonite

Clay minerals possess unique chemical and physical properties, such as a small particle size and the presence of charge on their surface, which gives them the ability to retain and release both organic and inorganic molecules. This could be the case for montmorillonite.

The hydration of clays involves the adsorption of water molecules on their surface, which can occur through hydration and interaction of the mineral surface with water molecules and with interlayer cations. The charges developed on the surface of the mineral (usually negative) [76] will promote the adsorption of cations, which behave as the main hydrophilic centres on the mineral. In this case, montmorillonite shows a much higher affinity for the zwitterionic (81%) form than for the anionic (19%) form since the positive charge is missing. Previous work on other amino acids, such as glycine adsorbed onto nontronite, also shows the same behaviour; amino acids are adsorbed on clays mainly in the zwitterionic form, although the anionic form is also sorbed but in a minor amount [77]. These results are consistent with the findings of our current study.

**Figure 5:**
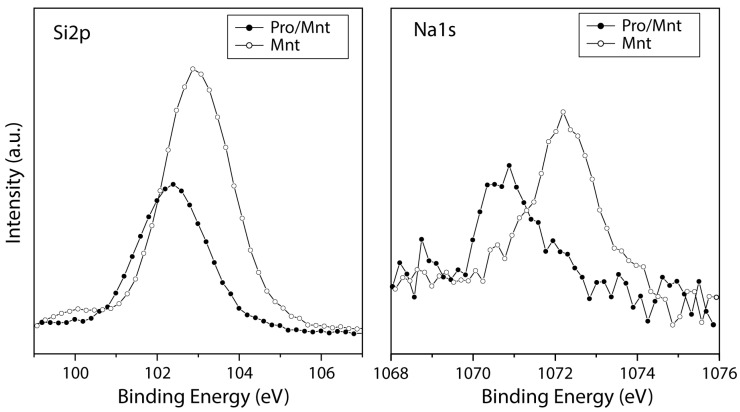
XPS spectra from the Si2p and Na1s regions for the montmorillonite mineral surface (empty circles) and after proline molecular adsorption on the mineral (black circles).

Figure 5 shows the spectrum of silicon (Si2p), the main chemical element in the montmorillonite structure, with a binding energy at 102.9 eV (assigned to the silicate species). After molecular adsorption, the intensity of the spectrum decreases, which can be due to amino acid adsorption on the surface, which attenuates the signal of the mineral. The same behaviour is observed for sodium, as the signal decreases after proline adsorption.

It has been observed that the negative layer charge on the silicate framework is delocalized over Si, O, and Al ions, owing to the systematic shifts of photoelectron binding energies. The chemical shift of the Si2p binding energy to lower binding-energy values may result from an increase in the negative layer charge in the silicate framework because of Si^4+^ replacement by Al^3+^ and/or Fe^3+^ [78]. This was confirmed in our work, where the Si2p peak is shifted towards lower binding energy values after proline adsorption (see Figure 5). Therefore, the interaction process of the positively charged zwitterion form in the negatively charged mineral is favoured over the anionic form.

#### 3.2.2. Iron Disulphide

**Figure 6:**
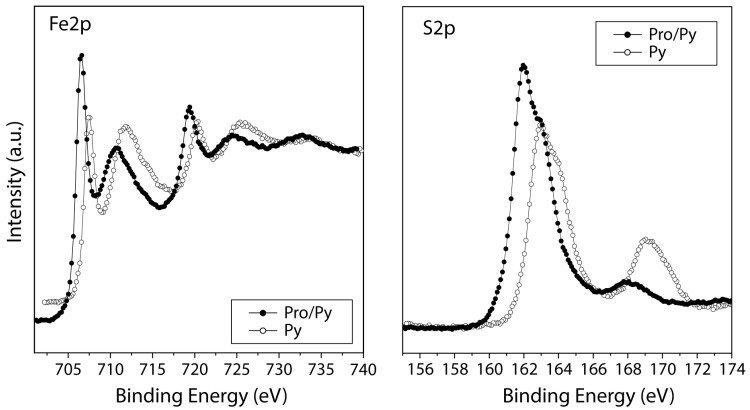
XPS spectra from the Fe2p and S2p regions for the iron disulphide mineral surface (empty circles) and after proline molecular adsorption on the mineral (black circles).

Figure 6 shows the XPS spectra comparison of the pyrite surface before and after the interaction of the molecules. It is clear that pyrite minerals in water solution are highly oxidized, which can be confirmed by the identification of both iron oxide and sulphate species from the Fe2p iron spectra at 709–711 eV FeO and Fe_2_O_3_. Furthermore, the sulphur S2p spectra show a peak at 168–169 eV characteristic of sulphate species, which confirms the oxidation process. The predominant component is iron disulphide at 706.7–707.7 eV (Fe2p region) and 162.0–163.0 eV (S2p region).

Under solution conditions, the pyrite surface is oxidized, so oxygen promotes an autocatalytic oxidation process on the pyrite surface. Proline anion adsorption has been detected on the oxidized surface, and zwitterion species adsorption is less significant under these conditions. Therefore, anion adsorption might compensate for the positive charge on the oxidized pyrite surface.

A comparison between the iron disulphide surface before and after molecular adsorption shows that iron oxide and iron disulphide species are similar in intensity to the isolated mineral, whereas in Pro/Pyrite systems, the component assigned to iron disulphide is predominant over iron oxides, which means that L-proline partially inhibits surface oxidation during the adsorption process. However, the clear identification of the iron oxide species present in both cases suggests that the interaction of the anionic form is favoured over that of the zwitterion.

#### 3.2.3. Haematite

**Figure 7:**
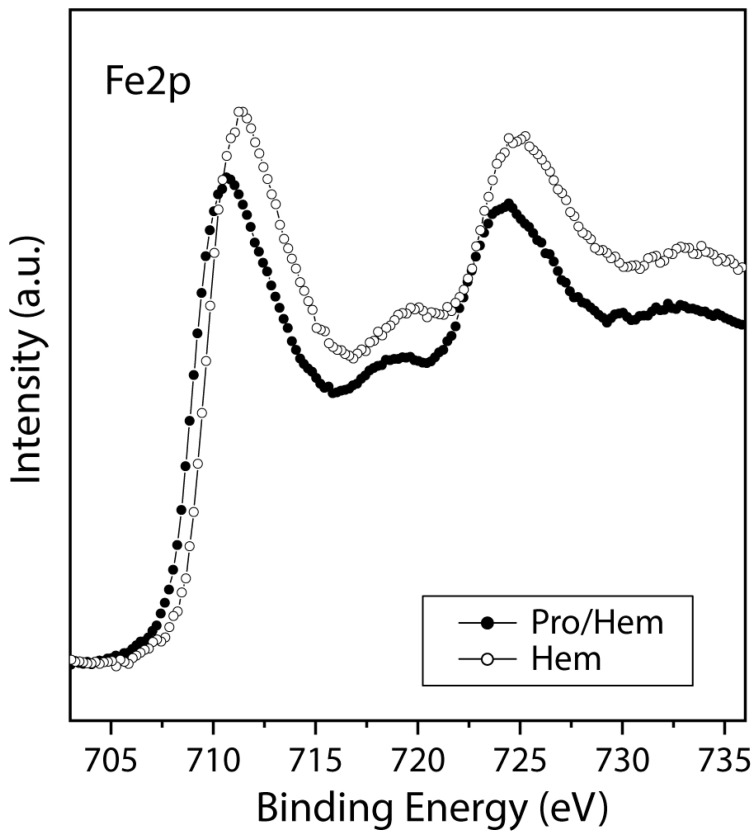
XPS spectra from the Fe2p region for the haematite mineral surface (empty circles) and after proline molecular adsorption on the mineral (black circles).

Figure 7 shows the XPS spectra comparison of the haematite surface before and after the interaction of the molecules. It is evident that the mineral haematite is the least reactive surface among the four tested in this study. First, the spectra of the N1s core level (a signature of the proline molecule), obtained from the comparative mineral study (Figure 1), show the lowest intensity for haematite. Second, the haematite surface after interaction with the proline solution does not show a noticeable decrease in the iron signal compared to the iron spectrum of the mineral alone. When molecular adsorption is successful, one would expect a noticeable decrease in the intensity of the initial surficial elements in the XPS spectrum, and this is not the actual case. However, even with a lower adsorption of proline than in any of the other adsorbed minerals, it can still be concluded that in the case of haematite, the anchoring of both species (zwitterionic and anionic) is equally favoured (Table 2). It seems that the positive (zwitterionic) selection effect as a silicate is compensated by the effect of the oxidation of iron in solution and therefore the need for the presence of the anion, which could compensate for the positive charge on the oxidized iron surface.

#### 3.2.4. Basaltic Olivine

**Figure 8:**
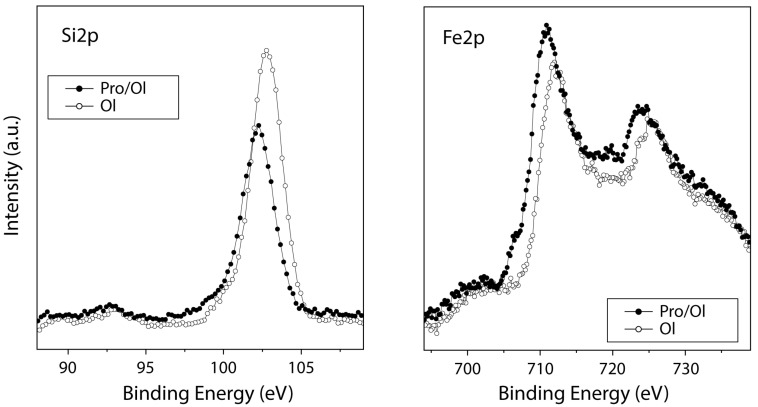
XPS spectra from the Fe2p and Si2p regions for the olivine mineral surface (empty circles) and after proline molecular adsorption on the mineral (black circles).

Figure 8 shows the spectra of silicon (Si2p) and iron (Fe2p), both chemical elements present in the structure of basaltic olivine, the former with a binding energy at 103.2 eV assigned to the silicate species and the latter at 710.7 eV assigned to iron oxides. After molecular adsorption, the intensity of the silicon spectrum slightly decreases, while that of iron is hardly attenuated at all; this result means that the proline molecule has a preferential affinity for silicon rather than iron atoms. Furthermore, low adsorption occurs on the surface of the olivine mineral, as depicted previously in the comparative mineral study (see Figure 1).

In this case, olivine shows a similar shift behaviour in the spectrum from 103.2 to 102.7 eV for Si2p towards lower binding energies after proline adsorption; this phenomenon was already described for montmorillonite. Thus, the interaction process of the positively charged zwitterion on the negatively charged mineral is somewhat favoured over the pyrite surface case. The presence of iron oxide species on the surface seems to compensate for the interaction inducing the coexistence of both chemical forms on the surface in almost equal percentages.

**Figure 9:**
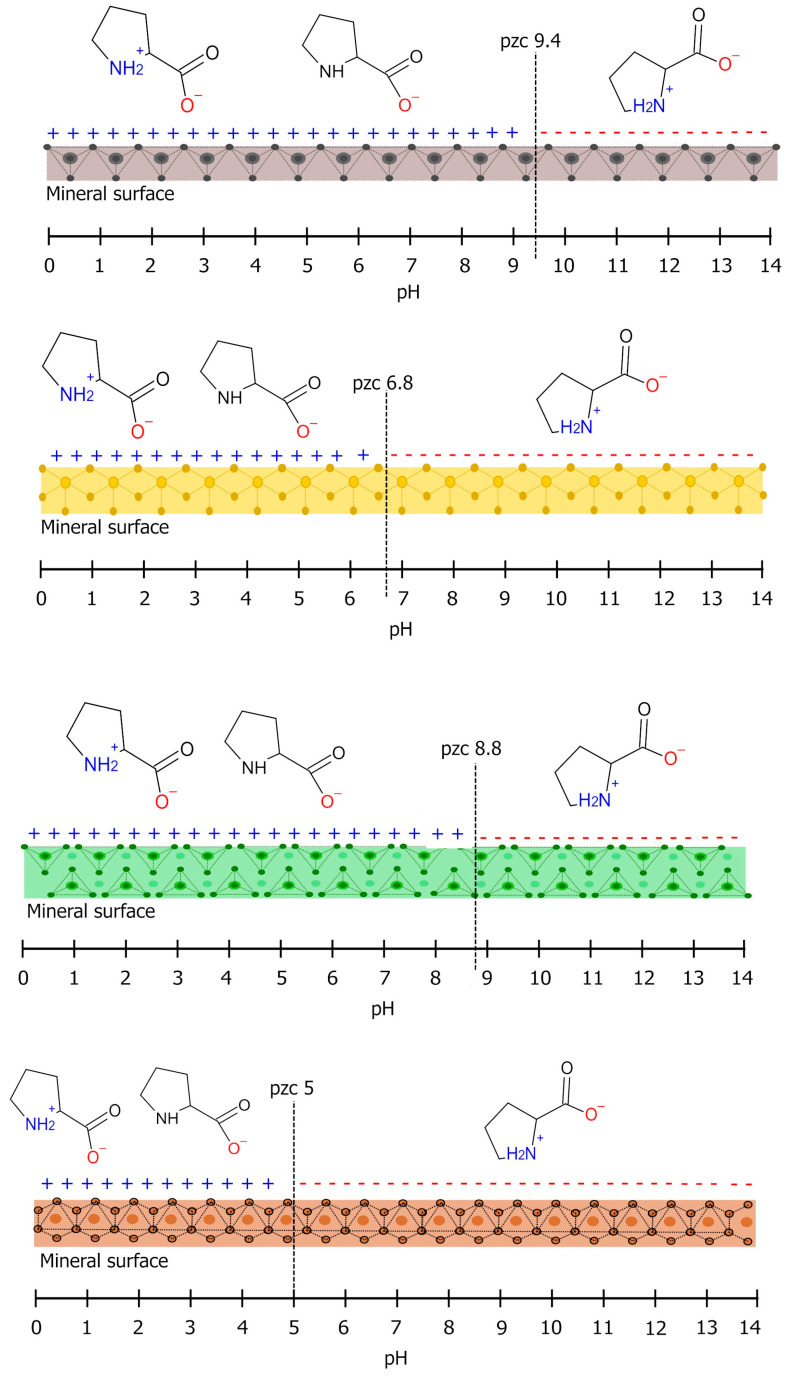
The Scheme shows a summary of the interactions between L-proline (anion or zwitterion) and the different mineral surfaces tested: (from top to bottom) montmorillonite, pyrite, olivine, and haematite.

A schematic representation of the different chemical forms of proline molecules interacting with different mineral surfaces has been made. Therefore, Figure 9 shows the possible interaction among the species of the amino acid (anion or zwitterion) and the surface. The point of zero charge (pzc) is the pH for which the net surface charge of adsorbent is equal to zero; then, pzc values were taken from different sources: montmorillonite, pyrite, olivine, and haematite [79,80,81,82].

Amino acid/mineral systems, relevant in prebiotic chemistry, have been studied from a new approach using complementary spectroscopic techniques (XPS and IR) and a diffraction technique (XRD) to study in detail the mineral phase instead of the solution. It is noteworthy to explain that XPS is not a routine technique in this field, but from our studies, we can probe its potential to study the molecular affinity, the surface sites of preference, the favoured chemical form of interaction, and the spectroscopic fingerprint to follow for molecular preservation. Therefore, the information extracted from our studies allows us to propose montmorillonite as a highly reactive mineral with an enormous capacity for molecular preservation, which would be more likely to be conducive to polymerization/condensation reactions. Furthermore, it preferentially selects the zwitterion form, which provides two crucial and reactive charge functional groups (COO^−^ and NH_2_) and thus two molecular sites to drive molecular chemistry and anchor possible surface reactions, depending on the surrounding conditions. Minerals played a crucial role in the origin of life and in prebiotic chemistry, but it is relevant to open up these studies to tools from other disciplines such as surface science to have a broader view and deeper understanding of surface chemistry, and thus to elucidate interesting implications for discerning catalysed prebiotic chemistry reactions.

## 4. Conclusions

We have performed the first spectroscopic characterization of L-proline adsorption on a battery of mineral surfaces, which is relevant to prebiotic chemistry. XPS analysis is used to accurately explore each molecular adsorption process and to determine the nature of the interaction (chemi/physisorption) of L-proline. In XPS spectroscopy, changes in the spectra of key elements in minerals reveal the interaction of organics with these surfaces. Noticeable differences in these spectra before and after adsorption dictate the mineral affinity towards one of the two preferable molecular forms of L-proline, carboxylate (anionic), or zwitterionic. Montmorillonite has a negative surface charge and shows preferential adsorption of the amino acid zwitterion, that is, a molecule related to the amino group (NH_2_^+^) that is positively charged. Iron disulphide has a surface that is easily oxidized, which implies the generation of a positive surface charge, favouring the sorption of the anionic form of proline. However, for olivine and haematite, there is a less remarkable preference for the adsorption of one of the chemical species.

Olivine is a magnesium iron silicate, where the positive iron and the negative silicate share surficial environments, and this mineral exhibits a slight preference for proline anion adsorption. In addition, haematite is the only mineral tested with no preferential adsorption of anionic or zwitterionic forms.

The understanding of the specific elemental role of the different mineral surfaces is of interest because it governs the interaction between them and the biomolecule of study. The XPS spectra of the mineral constituent elements show remarkable differences before and after molecular adsorption, proving the success of the molecule’s adsorption on the mineral surface. Indeed, XPS spectroscopy demonstrated which chemical form has been anchored, and in this case, the affinity towards one of the two preferred molecular forms of L-proline (anionic or zwitterionic) can be established.

In this research, specific mineral-organic interactions have been described; the adsorbed chemical forms and their charge could force the functional groups more kindred to the mineral surface, thus leaving the groups less reactive with the mineral free and available to activate chemical reactions with other molecules, leading to an increase in chemical complexity. Here, we have examined the physical processes and driving forces of the molecular interaction of four systems, amino acid (proline)/minerals, which will provide insight into the understanding of a wide range of chemical environments that would be prebiotically viable by concentrating precursor molecules and their subsequent reactivity due to the specific structure of each mineral. In addition, it is important to search for minerals with the highest molecular concentration potential, as well as those capable of protecting the molecules in their structure from UV damage. Therefore, the location of these minerals would be the planetary sites most likely to detect organic molecules. This study confirms the potential of clays such as montmorillonite to preserve and concentrate simple biomolecules such as amino acids, as well as pushing the chemistry governing molecular interaction.

## Figures and Tables

**Table 1:** Minerals employed in this study and their characteristics.

Mineral	Chemical Formula	Mineral Group	Environment
Montmorillonite ^1^	(Na,Ca)_0.3_(Al,Mg)_2_Si_4_O_10_(OH)_2_ ·nH_2_O	Phyllosilicate	Sedimentary, hydrothermal, diagenetic
Olivine ^2^	(Mg,Fe)_2_SiO_4_	Silicate	Basic and ultra-basic igneous rocks, Group species name
Pyrite ^1^	FeS_2_	Sulphide	Sedimentary, magmatic, metamorphic, and hydrothermal deposits
Haematite ^1^	Fe_2_O_3_	Oxide	Magmatic, hydrothermal, metamorphic, and sedimentary

^1^ Mineral species [56], ^2^ Mineral group [57].

**Table 2:** Summary of the proline species percentages adsorbed on each molecule/mineral studied system.

System of Study	Anion	Zwitterion	Mineral Composition
Pro/Mnt	19%	81%	Mg, Ca, Na silicate
Pro/Iron disulphide	65%	35%	Iron disulphide
Pro/Ol	57%	43%	Fe Mg silicate
Pro/Hem	50%	50%	Iron oxide

**Table 3:** List of peak assignments on the FT-IR spectra.

Wavenumber (cm^−1^)		Vibration Mode	Refs.
Mnt	Pro/Mnt		
3625	3614	ν(OH)	[68,69]
-	3069	ν_as_(CH_2_)	[69]
-	2981	ν_s_(CH_2_)	[69]
1633	-	ν(H-O-H)	[68]
-	1611	ν(COO^−^)	[69]
-	1559	δ(NH_2_^+^)	[69]
-	1407	ν(COO^−^)	[69]
-	1372	ν(C-H)	[69]
1114	1115	ν(Si-O) out of plane	[70]
1000	1007	ν(Si-O) in plane	[70]
797	796	Platy form of tridymite	[70]
**Hem**	**Pro/Hem**		
-	1611	ν(COO^−^)	[69]
-	1549	δ(NH_2_^+^)	[69]
-	1405	ν(COO^−^)	[69]
-	1376	ν(C-H)	[69]
1089	1082	ν(SiO_4_) ^1^	[71,72]
1047	1030	ν(Si-O-Si) ^1^	[73]
908	903	ν(Si-OH) ^1^	[73]
799	796	ν(Si-O-Si) ^1^	[73]
518	519	δ(Fe-O)	[73]
441	441	δ(Fe-O)	[73]
**Ol**	**Pro/Ol**		
-	3068	ν_as_(CH_2_)	[69]
-	2975	ν_s_(CH_2_)	[69]
-	1612	ν(COO^−^)	[69]
-	1559	δ(NH_2_^+^)	[69]
-	1449	ν(COO^−^)	[69]
-	1379	ν(C-H)	[69]
1086	1087	ν(Si-O)	[70]
959	959	ν(SiO_4_) tetrahedron structure	[71,72]
929	930	ν(SiO_4_) tetrahedron structure	[71,72]
740	743	ν(SiO_4_) tetrahedron structure	[71,72]

^1^ Quartz impurities.

## Data Availability

Not applicable.
